# Molecular Mechanism of Chronic Viral and Non-Viral Liver Diseases

**DOI:** 10.3390/ijms24076218

**Published:** 2023-03-25

**Authors:** Tatsuo Kanda

**Affiliations:** Division of Gastroenterology and Hepatology, Department of Medicine, Nihon University School of Medicine, 30-1 Oyaguchi-kamicho, Itabashi-ku, Tokyo 173-8610, Japan; kanda.tatsuo@nihon-u.ac.jp; Tel.: +81-3-3972-8111

In this Special Issue, “Molecular Mechanism of Chronic Viral and Non-viral Liver Diseases”, invaluable articles have been published. 

Alcohol-associated liver disease (ALD) is one of the major liver diseases, and alcohol promotes hepatic fibrosis and hepatocarcinogenesis [1]. During the COVID-19 pandemic, alcohol consumption has continued to rise [2], and age-standardized mortality rates for ALD increased during the COVID-19 pandemic in the United States [3]. In Japan, the COVID-19 epidemic might be associated with an increase in hospital admissions by ALD and alcohol-related pancreatitis [4]. Sasaki-Tanaka et al. reviewed the molecular mechanism associated with hepatocarcinogenesis in ALD [1]. Alcohol oxidation, acetaldehyde, hepatic steatosis, oxidative stress, and peroxidation are associated with hepatic injuries [1]. The immune mechanism also plays a role in the development of ALD. Host genetic factors are possibly involved in the development of alcoholic hepatic fibrosis and hepatocarcinogenesis. Risky alcohol consumption, such as lifetime risky drinking (>2 drinks daily) and single-occasion risky drinking (>4 drinks on one occasion), is of concern among those with a mental health condition and requires attention at an individual and population level [5].

Hepatitis B virus (HBV) infection causes acute and chronic hepatitis, cirrhosis, and hepatocellular carcinoma (HCC). Oncogenic potential of HBV is associated with the X gene product (HBx), which may be able to activate a cellular transcription factor at both viral and cellular promotor sequences in hepatocytes [6]. HBx may be a regulatory protein associated with HBV replication [7]. Ma et al. screened 1018 FDA-approved compounds and measured HBx-binding ability using a surface plasmon resonance imaging (SPRi) screening assay. Finally, they found that the tryptophan derivative, tranilast, inhibited HBV infection using human hepatocytes. Local catabolism of the amino acid tryptophan by indoleamine 2,3-dioxygenase (IDO) also seems an important mechanism of regulating T cell immunity [8]. SPRi screening assay may also be useful for investigating the molecular mechanism of drug development, as well as other modalities, such as in silico molecular docking or reverse LIGHTHOUSE analysis [9,10,11].

Qiu et al. examined the role of autoantibodies to paired box protein Pax-5 (PAX5), protein patched homolog 1 (PTCH1), and guanine nucleotide-binding protein subunit alpha-11 (GNA11) in the sera of Hispanic American patient with HCC [12]. They concluded that these autoantibodies to tumor-associated antigens may enhance the detection of HCC. Kim et al. reported a patient with Dubin–Johnson syndrome, who had a missense mutation in exon 18 of *adenosine triphosphate-binding cassette subfamily C member 2 (ABCC2)* [13].

Hepatic steatosis is common among patients scheduled for bariatric surgery, and steatohepatitis and liver fibrosis have been reported as potential complications of traditional jejunoileal bypass surgery [14]. Kalinowski et al. also reported that *mitochondrial amidoxime reducing component 1 (MTARC1)* rs2642438 and *hydroxysteroid 17-beta dehydrogenase 13 (HSD17B13)* rs72613567 polymorphisms had protective effects on nonalcoholic fatty liver disease (NAFLD)/nonalcoholic steatohepatitis (NASH) [15], although functions of these gene-coding proteins should be further explored.

The prevalence of NASH in morbidly obese patients was extremely high, and early intervention is recommended [16]. Bariatric surgery is useful for ensuring the long-term treatment of NAFLD/NASH in morbidly obese patients, and may be a therapeutic option for patients with obesity and type 2 diabetes mellitus resistant to conventional treatment [17,18]. Further studies are needed concerning the association between bariatric surgery and hepatic steatosis. Bariatric (metabolic) surgery improves hepatic steatosis and the histological lesions of metabolic-associated fatty liver disease, including fibrosis [19].

Interferon-free direct-acting antivirals against hepatitis C virus (HCV) are readily available, and their efficacy is higher, with fewer adverse events and a shorter duration, resulting in the decrease of HCV-related hepatic diseases; although, there are several problems [20]. However, there are still many unresolved issues of liver diseases, including chronic viral and non-viral hepatic diseases. In the new normal life, the molecular mechanism for chronic viral and non-viral hepatic diseases should be investigated.
